# Determination of band alignment in the single-layer MoS_2_/WSe_2_ heterojunction

**DOI:** 10.1038/ncomms8666

**Published:** 2015-07-16

**Authors:** Ming-Hui Chiu, Chendong Zhang, Hung-Wei Shiu, Chih-Piao Chuu, Chang-Hsiao Chen, Chih-Yuan S. Chang, Chia-Hao Chen, Mei-Yin Chou, Chih-Kang Shih, Lain-Jong Li

**Affiliations:** 1 Institute of Atomic and Molecular Sciences, Academia Sinica, No. 1, Roosevelt Road, Sec. 4, Taipei 10617, Taiwan.; 2 Department of Physics, University of Texas at Austin, Austin, Texas 78712, USA.; 3 National Synchrotron Radiation Research Center, Hsinchu 30076, Taiwan.; 4 School of Physics, Georgia Institute of Technology, Atlanta, Georgia 30332, USA.; 5 Deapartment of Physics, National Taiwan University, Taipei 10617, Taiwan.; 6 Physical Sciences and Engineering Division, King Abdullah University of Science and Technology, Thuwal 23955-6900, Kingdom of Saudi Arabia.

## Abstract

The emergence of two-dimensional electronic materials has stimulated proposals of novel electronic and photonic devices based on the heterostructures of transition metal dichalcogenides. Here we report the determination of band offsets in the heterostructures of transition metal dichalcogenides by using microbeam X-ray photoelectron spectroscopy and scanning tunnelling microscopy/spectroscopy. We determine a type-II alignment between MoS_2_ and WSe_2_ with a valence band offset value of 0.83 eV and a conduction band offset of 0.76 eV. First-principles calculations show that in this heterostructure with dissimilar chalcogen atoms, the electronic structures of WSe_2_ and MoS_2_ are well retained in their respective layers due to a weak interlayer coupling. Moreover, a valence band offset of 0.94 eV is obtained from density functional theory, consistent with the experimental determination.

## Abstract (web summary)

The alignment of the bandgap of adjacent materials in a heterostructure largely determines the electronic properties of a device. Here, the authors measure the conduction and valance band offsets at the interface between two two-dimensional materials: molybdenum disulphide and tungsten diselenide.

## Graphical abstract

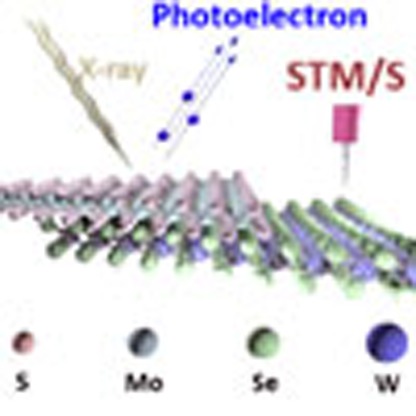

Transition metal dichalcogenides (TMDs) have emerged as a new platform for atomic layer electronics^1^ and optoelectronics^2^,^3^,^4^,^5^. Many proposed novel devices are based on heterostructures formed between dissimilar TMDs^6^,^7^,^8^,^9^,^10^,^11^,^12^,^13^. Heterojunction band offset is the key parameter for designing HJ-based electronic/photonic devices and accurate determination of this parameter is of critically important. In conventional semiconductor heterojunctions (HJs), one commonly used technique to determine the valence band offset (VBO) is XPS^14^,^15^,^16^,^17^. This method relies on finding the core-level alignment of two constituent semiconductors across the HJ. Then with additional information on the core-level position relative to the valence band maximum (VBM) measured separately for individual semiconductors, the VBM alignment across the HJ can be determined. The application of this technique to TMD HJs, however, faces two technical challenges. First, the HJ is formed only locally with a lateral lengthscale of only a few microns, owing to the limited lateral size of available TMD monolayer samples. It is thus necessary to locate such locally formed HJs and measure the core-level alignment across the junction. The second challenge is the determination of the VBM position. In conventional semiconductors, the valence bands primarily comprise of *sp* orbitals, with a smooth density of states (DOS). This allows for a precise determination of the VBM through curve fitting. In single-layer (SL) TMDs, the DOS near VBM has a complicated line shape due to the different characteristics of the states near Γ and K.

In the following, by using microbeam X-ray photoelectron spectroscopy (μ-XPS) where the photon spot can be focused down to sub-microns (spot size ∼100 nm), we are able to measure the core-level alignment across the TMD HJs at the local scale. Moreover, by using scanning tunnelling microscopy/spectroscopy (STM/S) to measure individual TMDs we determine the quasi-particle gaps and the fine structures involved, such as the energy difference between the VBM at Γ and K. The special combination of μ-XPS and STM/S measurements allow us to deduce the precise band offset values including both the VBO and conduction band offset (CBO). In addition, the first-principles calculations are performed to elucidate the interlayer interaction.

## Results

### The μ-XPS measurements

For μ-XPS measurements, the single-crystalline monolayer TMDs were synthesized using chemical vapour deposition on sapphire substrates^18^,^19^,^20^. These as-grown TMDs were then detached from sapphire substrates and transferred onto Si wafers with native oxides to form the HJ stacks (see the Methods section). It has been shown recently that with the presence of a thin native oxide (∼2 nm) layer the photoemission measurement can be carried out without the charging effect, but the oxide is also thick enough to ensure that the Si band structure is suppressed so the valence band structure detected is only from SL-TMDs^21^. For STM measurements, SL-TMDs are grown on highly oriented-pyrolytic-graphite (HOPG) directly to provide enough conductivity for STM imaging.

**Figure 1:**
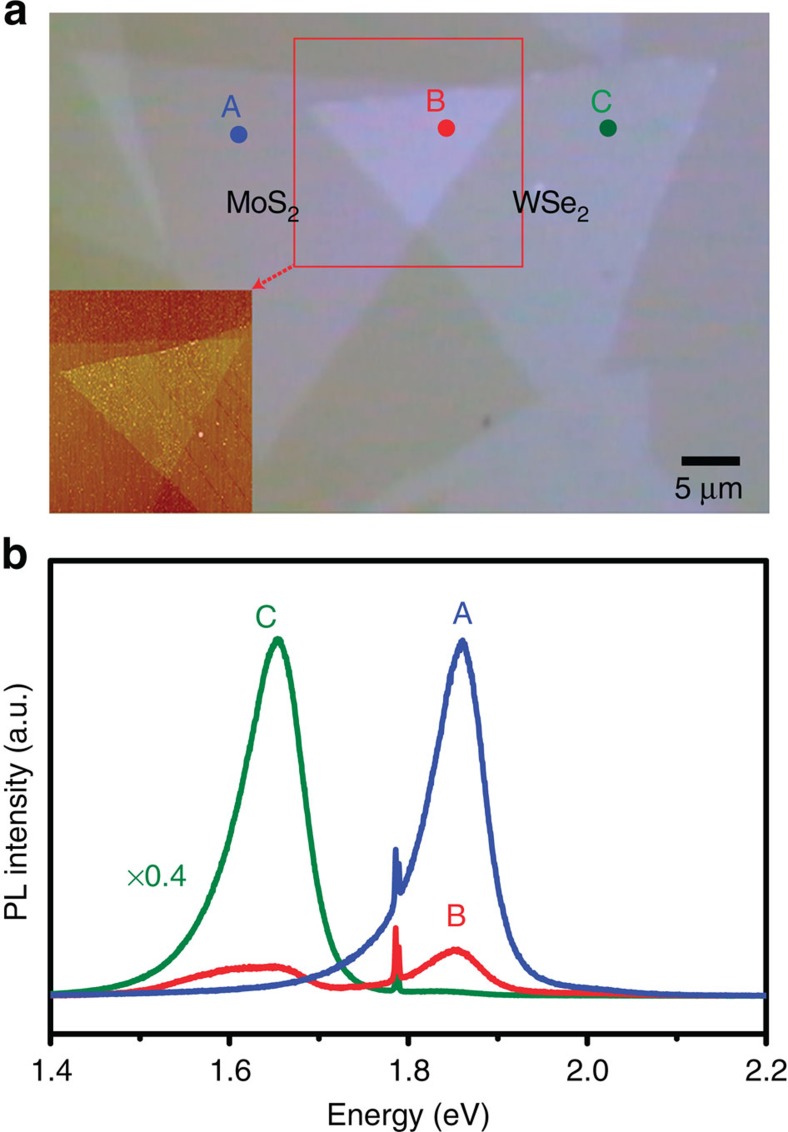
Optical microscopy images and photoluminescence spectroscopy taken on the staked WSe_2_/MoS_2_ heterostructure. (**a**) Optical micrograph and atomic force microscopy images for the WSe_2_/MoS_2_ heterostructural stacked flakes on a sapphire substrate. (**b**) Photoluminescence spectra for the selected sites including MoS_2_ only (A), WSe_2_ only (C) and WSe_2_/MoS_2_ (B) stacked areas.

Figure 1a shows the optical micrograph and atomic force microscopy images for the WSe_2_/MoS_2_ heterostructure stacked on a sapphire substrate. The characterizations by atomic force microscopy, optical gap measurements and Raman spectroscopy (Supplementary Fig. 1 and Supplementary Table 1) indicate that they are single-layer TMDs flakes^22^,^23^. Figure 1b shows the photoluminescence (PL) spectra for the selected sites including MoS_2_ only (A), WSe_2_ only (C) and WSe_2_/MoS_2_ (B) areas. The PL intensity of both MoS_2_ and WSe_2_ for the overlapped area (B) is significantly lower than that from WSe_2_ or MoS_2_ alone, indicating that the photoexcited carriers are quenched through other routes than the emission from individual WSe_2_ or MoS_2_ band edges. The surface adsorbates on these two-dimensional (2D) materials are removed by vacuum annealing at an elevated temperature^23^. Herein, these flakes are annealed in a high vacuum chamber (2 × 10^−10^ Torr) at 300 °C for over 8 h before the scanning photoelectron microscopy (SPEM) scans.

**Figure 2:**
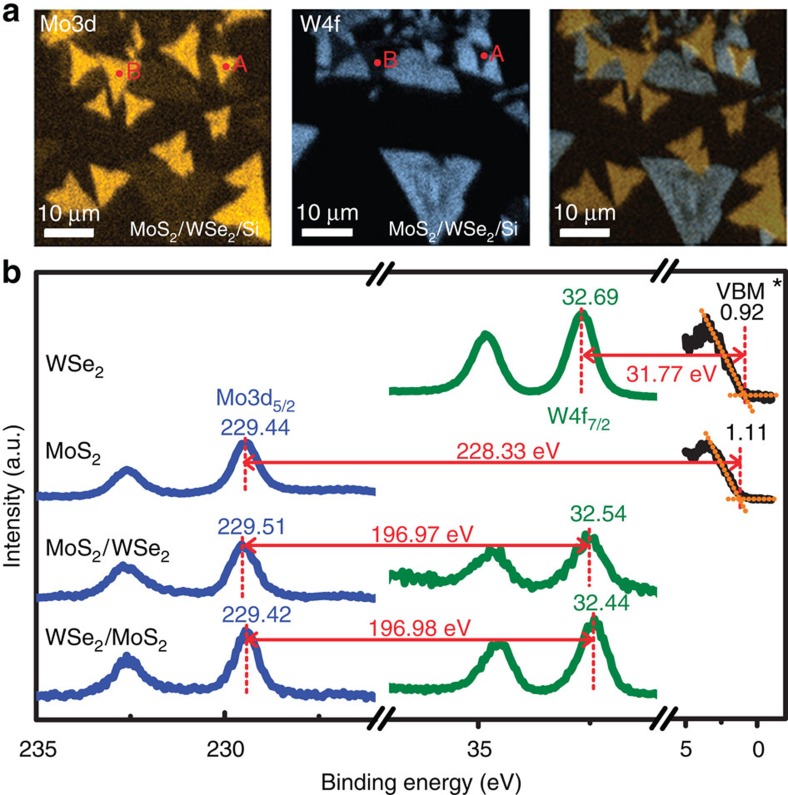
μ-XPS measurements on the stacked MoS_2_/WSe_2_ and WSe_2_/MoS_2_ heterostructures. (**a**) The Mo3d and W4f mappings for the same physical area. The right figure is the overlapped mapping that allows the identification of MoS_2_/WSe_2_ stacked areas. A and B points are the typical stacking area where the XPS are taken. (**b**) The spectra for the selected isolated WSe_2_ and MoS_2_ flakes, the stacked MoS_2_/WSe_2_ and WSe_2_/MoS_2_ heterostructure. All numbers are quoted in electron volt. There is an uncertainty of ±0.04 eV when determining the energy levels for all peaks.

Figure 2a shows the spatial mapping of Mo3d_5/2_ (left), W4f_7/2_ (middle) and their composite (right) respectively using μ-XPS. Such an elemental mapping allows us to identify isolated SL-WSe_2_, SL-MoS_2_ and locally stacked HJ bilayer unambiguously. The corresponding core-level spectra of W4f and Mo3d in isolated SL-WSe_2_, isolated SL-MoS_2_, the WSe_2_/MoS_2_ stack and the MoS_2_/WSe_2_ stack are shown in Fig. 2b. Also shown are the corresponding valence band (VB) spectra in isolated SL-WSe_2_ and SL-MoS_2_ flakes. The energy splittings and relative intensities due to the spin–orbit coupling for W4f (4f_7/2_ and 4f_5/2_) and Mo3d (3d_5/2_ and 3d_3/2_) doublets remain unchanged before and after forming 2D stacked films. Furthermore, their energy locations are independent of the X-ray beam flux, confirming the absence of the charging effect.

The core-level separation between the W4f_7/2_ and Mo3d_5/2_ is determined to be 196.97±0.04 eV across the HJ. In reverse stacking (that is, WSe_2_/MoS_2_), this value becomes 196.98±0.04 eV, essentially unchanged within the experimental error. To determine the VBO, we also need the respective quantities of core level relative to the VBM in individual SL-WSe_2_ and SL-MoS_2_. With a least-square fit of the leading edge of the VB spectra (shown in Fig. 2b), the apparent VBM locations are determined to be 0.92 eV for WSe_2_ and 1.11 eV for MoS_2_ (Supplementary Fig. 2). We recognize, however, that this fitting procedure may not lead to the correct value of VBM value due to the complicate line shape in the DOS (discussed further in Supplementary Note 1). We thus label this ‘apparent' VBM position as VBM*. Using this procedure, we determine that the Mo3d_5/2_ relative to the VBM* in MoS_2_ to be 228.33±0.04 eV and the W4f_7/2_ relative to the VBM* in WSe_2_ to be 31.77±0.04 eV. Assuming that the energy difference between the VBM* and the core level remains unchanged after stacking, the apparent VBO* for MoS_2_ and WSe_2_ and in the stacked film MoS_2_ on WSe_2_ can be determined as:


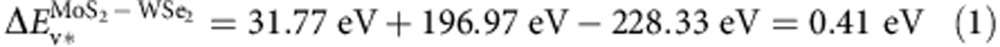


Here the notation MoS_2_−WSe_2_ in the superscript indicates that this is the potential step moving from MoS_2_ into WSe_2_. The error bar in the determination of the VBO* is estimated to be 0.07 eV. The consistent core-level separation of 196.98±0.04 eV between W4f and Mo3d in reverse stacking also implies the validity of VBO commutativity in TMD HJs. Following similar procedures, we determine the VBO* between WS_2_ and MoS_2_ as 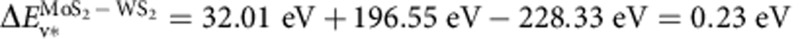 using the W and Mo core-level alignment; and the VBO* of WS_2_/WSe_2_ HJ as 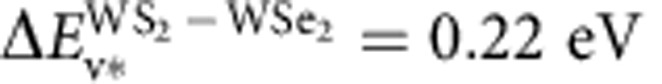 using the S and Se core-level alignment. One very interesting result is that the transitivity is satisfied. Namely VBO* (MoS_2_−WSe_2_) is essentially the same as VBO*(MoS_2_−WS_2_)+VBO*(WS_2_−WSe_2_) within the experimental error bar.

### Determination of band-edges by STM/S

Theoretical calculations show that the DOS near the VBM is dominated by the states near the Γ point with much less contribution from those near the K point where the true VBM is located (as shown in Supplementary Fig. 3b, and the generic band structures can also be seen in the Supplementary Fig. 3a.). Since XPS is unable to resolve the Γ-K splitting, the measured VBM* likely corresponds to the location of the Γ point. Thus, the resulting VBO* would not correspond to the true VBO. This shortcoming is overcome by using STS to accurately determine the quasi-particle gaps, and the Γ−K VBM energy splitting, Δ_Γ−K_. With these additional pieces of information, the band alignment (both valence band and conduction band) can be accurately determined.

**Figure 3:**
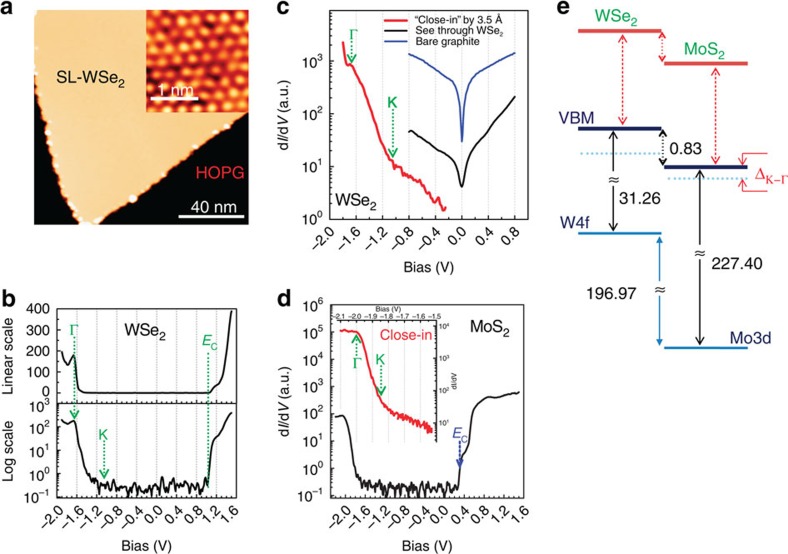
STM images and the tunnelling spectra of WSe_2_ and MoS_2_ grown on HOPG. (**a**) STM image of single layer WSe_2_ grown on HOPG. The inset shows the atomic resolution image taken on the SL-WSe_2_. For the inset, *U*=−1.4 V, I=10 pA. (**b**) The d*I/*d*V-V* spectrum taken on the SL-WSe_2_ flake. The tunnelling conductance d*I/*d*V* (with arbitrary unit) is plotted in both the linear scale (upper panel) and the logarithmic scale (lower panel). The green dashed arrows indicate the positions of the local VBM at Γ and K points, which are equal to −1.65 eV and −1.05 eV, respectively. The CBM is assigned at +1.03 eV. (**c**) The clear threshold, corresponding to VBM at K, can be seen in the d*I/*d*V* spectrum taken with much more close tip-sample distance (∼3.5 Å closer than **b**). For comparison, the spectra taken with the stabilization bias with the gap (−0.8 V) is shown in black, while the one taken on bare graphite is in blue. The similar d*I/*d*V* (in black) and ‘close-in' (red in inset) spectra of SL-MoS_2_ are displayed in **d**, while the valence band maxima at Γ and K are marked in the inset. The *E*_C_ (blue arrow) corresponds to the CBM. (**e**) The diagram of band alignments among single-layer MoS_2_ and WSe_2_. The local VBM at Γ and global VBM at K are shown in cyan and red, respectively.

Figure 3a shows the STM image of SL-WSe_2_ grown on HOPG, with a typical tunnelling spectrum shown in Fig. 3b. In the negative bias range, there is sharp peak at about −1.65 V then vanishes quickly as the bias is increased. In the positive sample bias range, an onset of conductivity occurs at +1.03 V. To determine the VBM and CBM positions, the conductivity is displayed in the logarithmic scale (lower panel in Fig. 3b) where the sharp onset at 1.03 V can be unambiguously identified as the CBM. The determination of the VBM using such a d*I/*d*V* spectrum, however, encounters some ambiguity. An extrapolation of the leading edge would yield a threshold being located somewhere between −1.4 and −1.3 V. Detailed examination, however, reveals that the global VBM at the K point is located at a significantly higher energy location (at −1.05 V) and the peak at the −1.65 V corresponds to the local VBM at the Γ point. In the WSe_2_ region, as long as the junction stabilization voltage (namely, the applied bias before the interruption of the feedback) is between −1.65 and −2.0 V, the spectrum does not have enough sensitivity to detect the global VBM position. To enhance the ability to observe these states, we acquire another spectrum by reducing the tip-to-sample distance by ∼3.5 Å, shown as the red curve in Fig. 3c. In this ‘close-in' distance, tunnelling from the states of the underlying graphite can also be detected. Moreover, there is a threshold at −1.05±0.05 V separating the states derived from graphite and those from WSe_2_. This threshold is assigned as the global VBM at the K point. By using a stabilization voltage of −0.8 V (well into the gap region of WSe_2_), the underlying graphite electronic states can be ‘seen through SL-WSe_2_' and the spectrum is essentially the same as the spectrum acquired in the bare graphite surface (shown as the black and blue curves in Fig. 3c). This also confirms our assignment of the threshold at −1.05 V to be the global VBM position at K point. More detailed discussions on the determination of energy location of different thresholds and their origins in the Brillouin Zone can be found in ref. 24. In Fig. 3d, we show a close-in spectrum for MoS_2_ where the Γ point can be identified at −2.0 V while the VBM is located at −1.84±0.05 eV. The CBM location of MoS_2_, can also be resolved as 0.31±0.05 eV.

To determine the core-level position relative to the true VBM in SL-TMD, we carry out μ-XPS on similarly prepared single-layer MoS_2_ and WSe_2_ on HOPG (Supplementary Fig. 4). The measurements yield a binding energy of 229.24±0.03 eV for Mo3d_5/2_, corresponding to a separation of 227.40 eV to the true VBM (at the K point) in MoS_2_. Similarly, W4f_7/2_ in SL-WSe_2_ on graphite has a binding energy of 32.31±0.03 eV, corresponding to a value of 31.26 eV when referenced to the true VBM. With these two binding energies relative to their individual VBM determined, we can reapply the same algorithm and obtain a true VBO of


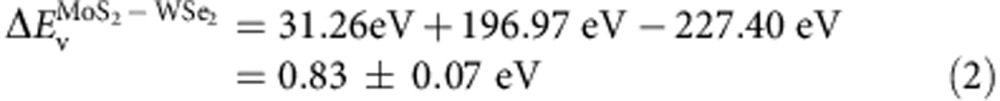


One can immediately see that the VBO measured is 0.42 eV larger than the apparent VBO* of 0.41 eV measured using XPS alone. This is due to the fact that in WSe_2_, the K point is 0.60 eV higher than the Γ point while in MoS_2_, it is only about 0.16 eV higher. With these STS energy data, the VBO* value can be deduced as 0.39 eV, very close to the XPS values of 0.41 eV. This affirms our earlier conjecture that the XPS measurement of VBM* is dominated by the high DOS at the Γ point. STS measurements of quasi-particle gaps (2.15±0.1 eV for MoS_2_ and 2.08±0.1 eV for WSe_2_) also allow us to deduce a CBO of 0.76±0.12 eV, affirming a type-II band alignment as illustrated in Fig. 3e.

Another very interesting observation is that for individual SL-MoS_2_ and WSe_2_ on graphite the binding energy difference between Mo3d_5/2_ and W4f_7/2_ is 229.24−32.31=196.93±0.04 eV, essentially the same as the core-level separation (196.97±0.04 eV) across the HJ stack. This means that ‘the supporting graphite substrate is a good common energy reference' for TMDs when we consider the problem of band alignment. In fact, if we take directly the VBM measured in STS for SL-TMD on graphite (−1.05 eV for WSe_2_ and −1.84 eV for MoS_2_), a VBO of 0.79 eV is deduced when we choose graphite as the energy reference. Why does this work? If we treat SL-TMD on graphite as a semiconductor–semimetal heterojunction, then the measured VBM position of SL-TMD on graphite also represents the VBO of the TMD/graphite heterojunction. If we then use the VBOs determined for SL-MoS_2_/graphite and SL-WSe_2_/graphite, and apply the transitivity we will deduce a VBO of 0.79 eV for MoS_2_/WSe_2_ heterojunction system, essentially the same as the VBO measured using XPS. We suggest that the reason this transitivity holds is related to the weak van der Waals interactions between TMDs and graphite and between different TMDs.

### First-principles calculations of the TMD HJs

**Figure 4:**
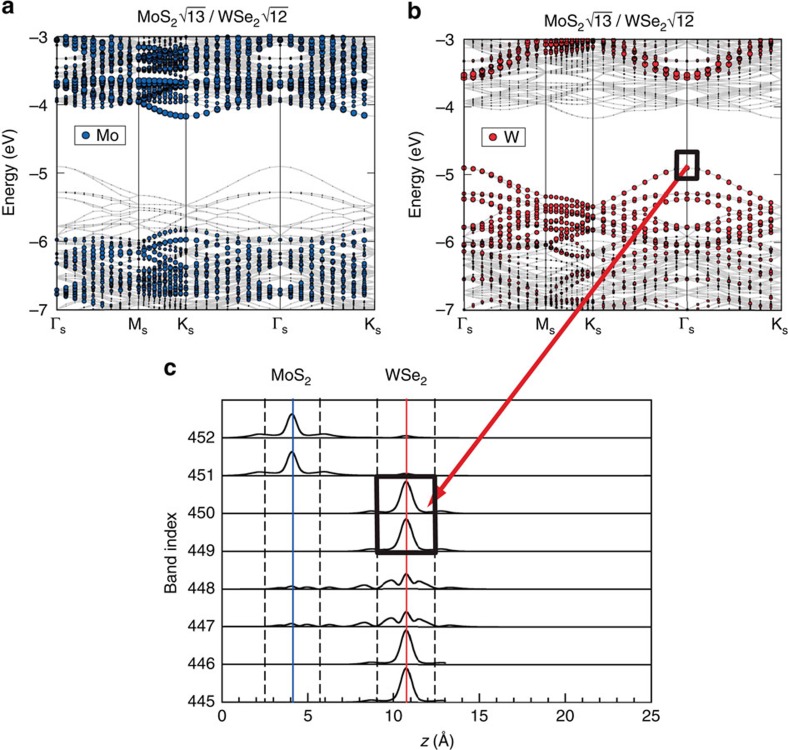
First-principles calculations for the electronic structures of the composite system formed by two dissimilar SL-TMD layers. (**a**,**b**), Energy band structure of the MoS_2_/WSe_2_ bilayer calculated using a supercell containing rotated 
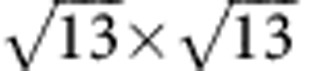
 and 
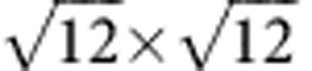
 unit cells of MoS_2_ and WSe_2_, respectively, to minimize the strain in individual layers due to lattice mismatch. The labelling of the symmetry points is referenced to the Brillouin zone of the supercell. Note that the original K point in the MoS_2_ SL remains at K_s_, while the original K point in the WSe_2_ SL is folded to Γ_s_. The projected bands onto Mo and W atoms are shown in **a**,**b**, respectively, with the amount of Mo (W) projection represented by the size of blue (red) circles. (**c**) One-dimensional charge density (integrated over the horizontal direction) illustrating that the states at the band edges belong to a distinct layer.

One fundamental question to address is whether or not individual layers retain their respective electronic structures even after stacking. The definition heterojunction would be meaningful only if this is true. To address this issue, we have carried out theoretical density functional theory (DFT) calculations for the electronic structures of the composite system formed by two dissimilar SL-TMD layers. Shown in Fig. 4 is the case for MoS_2_/WSe_2_ calculated using a 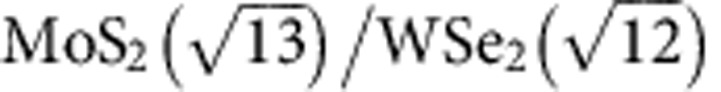 supercell to accommodate the difference in lattice constants. The direct bandgap at the original K point in the isolated MoS_2_ layer remains at K for the Brillouin zone of the supercell (labelled as *K*_s_ in Fig. 4), while the direct bandgap at the original K point in the WSe_2_ layer is folded to the Γ_s_ point. As can be seen, the electronic structures can be nicely projected into their respective MoS_2_ and WSe_2_ layers that are the same as the isolated layers, confirming the validity of treating the MoS_2_/WSe_2_ as a heterojunction. Moreover, a numerical value of 0.94±0.1 eV for the VBO is obtained in the DFT calculation which agrees very well with the 0.83±0.07 eV measured experimentally.

Theoretically, we have also carried out the calculations for heterostructures with other combinations of TMDs. The interlayer interaction between two TMD layers with the same chalcogen species turns out to be significant enough to change the characteristics of the states at the VBM. As an example, the MoS_2_/WS_2_ heterostructure is presented in Supplementary Note 2, and the energy bands are shown in Supplementary Fig. 5. We find that the band offset at the K point remains well defined and appears to be independent of the stacking pattern. However, the interlayer coupling moves the VBM position in the WS_2_ layer from K to Γ point, creating an indirect gap about 0.1–0.2 eV smaller than the direct gap^6^ (see Supplementary Fig. 5). Thus, for optical property that is dominated by the direct transition at the K point, the band offset concept may remain valid^6^,^25^, but one has to take into account the indirect bandgap in transport measurements.

## Discussion

In summary, by using μ-XPS, in conjunction with scanning tunnelling spectroscopy, we have shown the capability to determine the band alignment in locally formed TMD heterostructures. We determine a type-II alignment in WSe_2_/MoS_2_ with a VBO of 0.83±0.07 eV and a CBO of 0.76±0.12 eV. We further discover that the TMDs and the supporting graphite also form a semiconductor/semimetal heterostructure such that the transitivity hold for different heterostructures formed between SL-TMDs and TMD/graphite. Theoretical investigations show that the electronic band structure of the stacked TMD bilayers containing different chalcogen species resembles a superposition of the energy bands of individual layers, upholding the definition of semiconductor heterojunction. Quantitative agreement of VBO value is found between theoretical calculations and experimental measurements.

## Methods

### Growth and characterizations of 2D monolayers

The direct growth of MoS_2_ or WSe_2_ monolayer crystal flakes on a sapphire substrate by the vapour-phase reaction has been reported in our previous reports^19^,^20^. In brief, high-purity metal trioxides MO_3_ (M=Mo, W) was placed in a ceramic boat at the heating centre of the furnace. A sapphire substrate was placed in the downstream side adjacent to the ceramic boat. Sulfur or Selenium powder was heated by a heating tape and carried by Ar or Ar/H_2_ to the furnace heating centre. The furnace was then gradually heated from room temperature to desired temperature for reaction. After the reaction process, furnace was naturally cooled down to room temperature. These monolayers were characterized atomic force microscopy (Veeco Dimension-Icon system) and a confocal Raman /photoluminescence system (NT-MDT equipped with a 473-nm laser with the spot size of ∼0.5 μm).

### Scanning tunnelling microscopy

All STM investigations reported here were acquired at 77 K in ultra-high-vacuum (UHV; base pressure <6 × 10^−11^ Torr). Electrochemically etched W-tips were cleaned *in situ* with electron beam bombardment. The tunnelling bias is applied on sample. Before STM investigations, the samples were cleaned *in situ* by heating it up to 250 °C for an extended time (typically longer than 2 h). The conductance spectra were taken by using a lock-in amplifier with a modulation voltage of 10 mV and at a frequency of 724 Hz.

### Preparation of stacking layers

To stack the MoS_2_ (WSe_2_) monolayer flakes on the undoped Si substrate, a layer of PMMA thin film was coated on the as-grown MoS_2_ (WSe_2_) on sapphire as a transfer supporting layer^26^. After dipping in an aqueous NaOH (HF) solution, the PMMA-supported MoS_2_ (WSe_2_) monolayer was detached from sapphire substrates and transferred to the Si(111) substrate (Sheet resistance >300 Ohm sq^−1^), followed by the removal of PMMA using acetone. Various 2D heterostructural stacking films such as WSe_2_ on MoS_2_ or WSe_2_ on MoS_2_ can be obtained by transferring desired 2D flakes onto the other flake.

### Microbeam SPEM

The synchrotron radiation-based SPEM system is located at beam line 09A1 of National Synchrotron Radiation Research Center, Taiwan. The system is composed of a 16-channel hemisphere electron energy analyser (model 10–360, PHI), a sample scanning stage, and Fresnel zone plate optics to focus the monochromatic soft X-ray down to 100 nm. By synchronized raster scanning the sample relative to the focused soft X-ray, the excited photoelectrons (PE) were collected and analysed by the analyser. The PE intensity of specific core-level line can be converted into a two-dimensional surface chemical state distribution image^27^. SPEM can also be operated in μ-XPS mode to acquire high-resolution PE spectra. Combining the imaging and μ-XPS modes makes SPEM a suitable instrument to study the heterostructure of novel 2D materials^28^. The photon energy utilized in this study was 400 eV, which was calibrated every 8 h with the Au 4*f* core-level line of a clean gold foil electrically contacted with the samples to ensure the measurement reproducibility.

### Theoretical calculations

First-principles calculations were carried out within DFT using the Vienna *Ab initio* simulation package (VASP)^29^,^30^. The interactions between electrons and ions is described by the projector augmented wave method^31^, and the exchange-correlation potential is described by the Perdew–Burke–Ernzerhof-generalized gradient approximation^32^, with vdW corrections incorporated with vdW-DF functionals^33^. A supercell size of 25 Å is used to eliminate the spurious image interaction in the slab calculation, and the energy cutoff of plane waves is 600 eV. The lattice constants of monolayer MoS_2_ and WSe_2_ used in the simulation are 3.17 and 3.31 Å, respectively. The interlayer spacing is 6.67 Å between MoS_2_ and WSe_2_. More detail can be seen in Supplementary Note 3.

## Additional information

**How to cite this article:** Chiu, M.-H. *et al*. Determination of band alignment in the single-layer MoS_2_/WSe_2_ heterojunction. *Nat. Commun.* 6:7666 doi: 10.1038/ncomms8666 (2015).

## Supplementary Material

Supplementary Information Supplementary Figures 1-5, Supplementary Table 1, Supplementary Notes 1-3 and Supplementary References

## References

[b1] Radisavljevic B., Radenovic A., Brivio J., Giacometti V. & Kis A. Single-layer MoS_2_ transistors. Nat. Nanotechnol. 6, 147–150 (2011) . 21278752 10.1038/nnano.2010.279

[b2] Splendiani A. . Emerging photoluminescence in monolayer MoS_2_. Nano Lett. 10, 1271–1275 (2010) . 20229981 10.1021/nl903868w

[b3] Mak K. F., Lee C., Hone J., Shan J. & Heinz T. F. Atomically thin MoS_2_: a new direct gap semiconductor. Phys. Rev. Lett. 105, 136805 (2010) 136805 . 21230799 10.1103/PhysRevLett.105.136805

[b4] Wu S. F. . Electrical tuning of valley magnetic moment through symmetry control in bilayer MoS_2_. Nat. Phys. 9, 149–153 (2013) .

[b5] Xiao D., Liu G. B., Feng W. X., Xu X. D. & Yao W. Coupled spin and valley physics in monolayers of MoS_2_ and other Group-VI dichalcogenides. Phys. Rev. Lett. 108, 196802 (2012) . 23003071 10.1103/PhysRevLett.108.196802

[b6] Rivera P. . Observation of long-lived interlayer excitons in monolayer MoSe_2_–WSe_2_ heterostructures. Nat. Commun. 6, 6242 (2015) . 25708612 10.1038/ncomms7242

[b7] Tongay S. . Tuning interlayer coupling in large-area heterostructures with CVD-grown MoS_2_ and WS_2_ Monolayers. Nano Lett. 14, 3185–3190 (2014) . 24845201 10.1021/nl500515q

[b8] Lee C.-H. . Atomically thin p–n junctions with van der Waals heterointerfaces. Nat. Nanotechnol. 9, 676–681 (2014) . 25108809 10.1038/nnano.2014.150

[b9] Cheng R. . Electroluminescence and photocurrent generation from atomically sharp WSe_2_/MoS_2_ heterojunction p–n diodes. Nano Lett. 14, 5590–5597 (2014) . 25157588 10.1021/nl502075n PMC4189621

[b10] Hong X. P. . Ultrafast charge transfer in atomically thin MoS_2_/WS_2_ heterostructures. Nat. Nanotechnol. 9, 682–686 (2014) . 25150718 10.1038/nnano.2014.167

[b11] Fang H. . Strong interlayer coupling in van der Waals heterostructures built from single-layer chalcogenides. Proc. Natl Acad. Sci. USA 111, 6198–6202 (2014) . 24733906 10.1073/pnas.1405435111 PMC4035947

[b12] Britnell L. . Strong light-matter interactions in heterostructures of atomically thin films. Science 340, 1311–1314 (2013) . 23641062 10.1126/science.1235547

[b13] Withers F. . Light-emitting diodes by band-structure engineering in van der Waals heterostructures. Nat. Mater. 14, 301–306 (2015) . 25643033 10.1038/nmat4205

[b14] Franciosi A. & Van de Walle C. G. Heterojunction band offset engineering. Surf. Sci. Rep. 25, 1–140 (1996) .

[b15] Bratina G., Sorba L., Antonini A., Biasiol G. & Franciosi A. AlAs-GaAs heterojunction engineering by means of Group-IV elemental interface layers. Phys. Rev. B 45, 4528–4531 (1992) . 10.1103/physrevb.45.4528 10002079

[b16] KowalczykS. P., CheungJ. T., KrautE. A. & GrantR. W. CdTe-HgTe 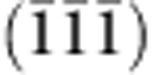 heterojunction valence-band discontinuity: a common-anion-rule contradiction. Phys. Rev. Lett. 56, 1605–1608 (1986) .1003272010.1103/PhysRevLett.56.1605

[b17] Shih C. K. & Spicer W. E. Determination of a natural valence-band offset: the case of HgTe- CdTe. Phys. Rev. Lett. 58, 2594–2597 (1987) . 10034792 10.1103/PhysRevLett.58.2594

[b18] Cong C. X. . Synthesis and optical properties of large-area single crystalline 2D semiconductor WS_2_ monolayer from chemical vapor deposition. Adv. Opt. Mater. 2, 131–136 (2014) .

[b19] Huang J. K. . Large-area synthesis of highly crystalline WSe_2_ mono layers and device applications. ACS Nano 8, 923–930 (2014) . 24328329 10.1021/nn405719x

[b20] Lee Y. H. . Synthesis of large-area MoS_2_ atomic layers with chemical vapor deposition. Adv. Mater. 24, 2320–2325 (2012) . 22467187 10.1002/adma.201104798

[b21] Jin W. C. . Direct measurement of the thickness-dependent electronic band structure of MoS_2_ using angle-resolved photoemission spectroscopy. Phys. Rev. Lett. 111, 106801 (2013) . 25166690 10.1103/PhysRevLett.111.106801

[b22] Tonndorf P. . Photoluminescence emission and Raman response of monolayer MoS_2_, MoSe_2_ and WSe_2_. Opt. Express 21, 4908–4916 (2013) . 23482024 10.1364/OE.21.004908

[b23] Zhang C. D., Johnson A., Hsu C. L., Li L. J. & Shih C. K. Direct imaging of the band profile in single layer MoS_2_ on graphite: quasiparticle energy gap, metallic edge states and edge band bending. Nano Lett. 14, 2443–2447 (2014) . 24783945 10.1021/nl501133c

[b24] Zhang C. D. . Probing critical point energies of transition metal dichalcogenides: surprising indirect gap of single layer WSe2. Preprint at http://arxiv.org/abs/1412.8487 (2014) . 10.1021/acs.nanolett.5b01968 26389585

[b25] van der Zande A. M. . Tailoring the electronic structure in bilayer molybdenum disulfide via interlayer twist. Nano Lett. 14, 3869–3875 (2014) . 24933687 10.1021/nl501077m

[b26] Lebegue S. & Eriksson O. Electronic structure of two-dimensional crystals from ab initio theory. Phys. Rev. B 79, 115409 (2009) .

[b27] Chiou J. W. X-rays in nanoscience: spectroscopy, spectromicroscopy, and scattering techniques Ch. 4 Weinheim : Wiley-VCH (2010) .

[b28] Shiu H. W. . Graphene as tunable transparent electrode material on GaN: layer-number-dependent optical and electrical properties. Appl. Phys. Lett. 103, 081604 (2013) .

[b29] Kresse G. & Furthmuller J. Efficiency of *ab-initio* total energy calculations for metals and semiconductors using a plane-wave basis set. Comp. Mater. Sci. 6, 15–50 (1996) . 10.1103/physrevb.54.11169 9984901

[b30] Kresse G. & Furthmuller J. Efficiency of ab-initio total energy calculations for metals and semiconductors using a plane-wave basis set. Comput. Mater. Sci. 6, 15–50 (1996) . 10.1103/physrevb.54.11169 9984901

[b31] Blochl P. E. Projector augmented-wave method. Phys. Rev. B 50, 17953–17979 (1994) . 10.1103/physrevb.50.17953 9976227

[b32] Perdew J. P., Burke K. & Ernzerhof M. Generalized gradient approximation made simple. Phys. Rev. Lett. 77, 3865–3868 (1996) . 10062328 10.1103/PhysRevLett.77.3865

[b33] Klimes J., Bowler D. R. & Michaelides A. Van der Waals density functionals applied to solids. Phys. Rev. B 83, 195131 (2011) .

