# Supplementary material for: Determination of band alignment in the single-layer MoS2/WSe2 heterojunction
Source: Nat Commun. 2015 Jul 16;6:7666. doi: 10.1038/ncomms8666 (PMC4518320; doi:10.1038/ncomms8666)
Supplement: Supplementary Information — Supplementary Figures 1-5, Supplementary Table 1, Supplementary Notes 1-3 and Supplementary References [file ncomms8666-s1.pdf]

Supplementary Figures

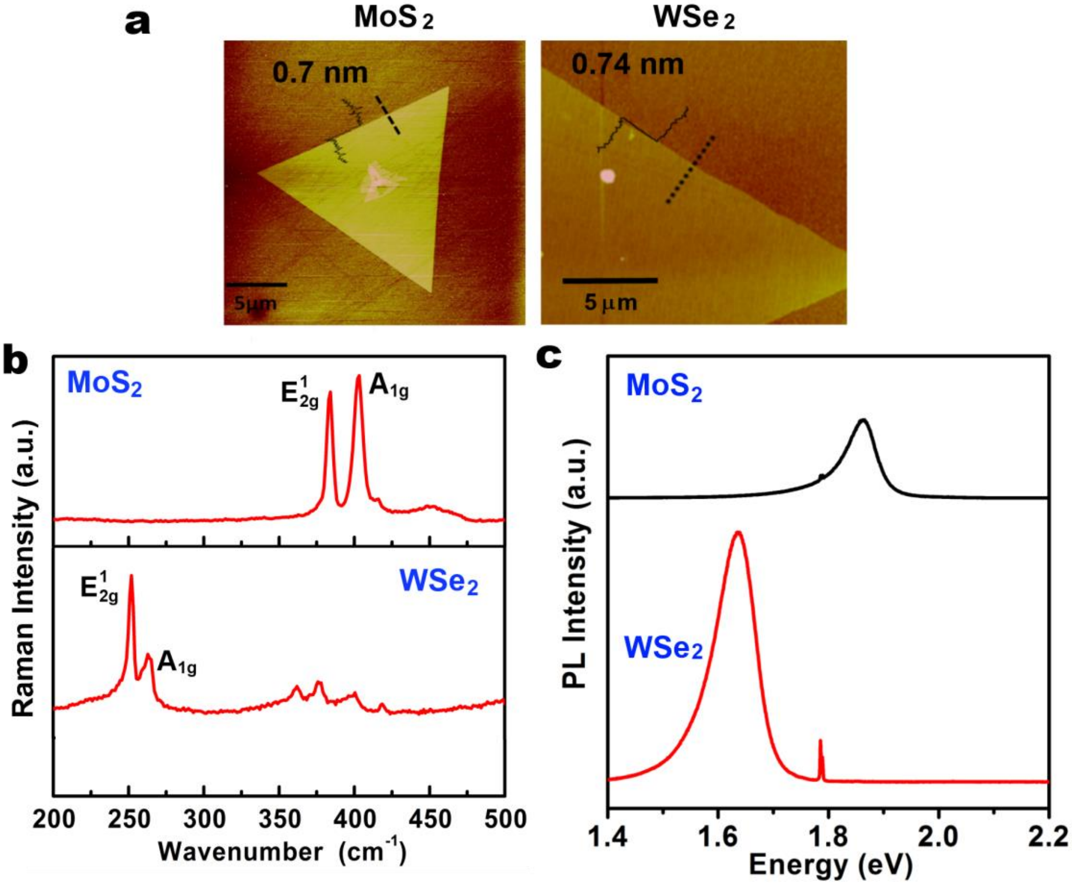

Supplementary Figure 1 | Characterizations of synthetic transition metal dichalcogenides monolayers on sapphire substrates. a, The thickness obtained from AFM cross-sectional profiles is around 0.7 to 0.8 nm, indicating that these as-grown flakes are single-layered. **b.** Raman and **c.** PL spectra for the synthetic MoS_2 and WSe_2 flakes. These spectroscopic features are consistent with those obtained from exfoliated single layers as shown in Supplementary Table 1. The measurements were performed in a confocal Raman/photoluminescence system equipped with a 473 nm laser with the spot size of ~ 0.5 μm.

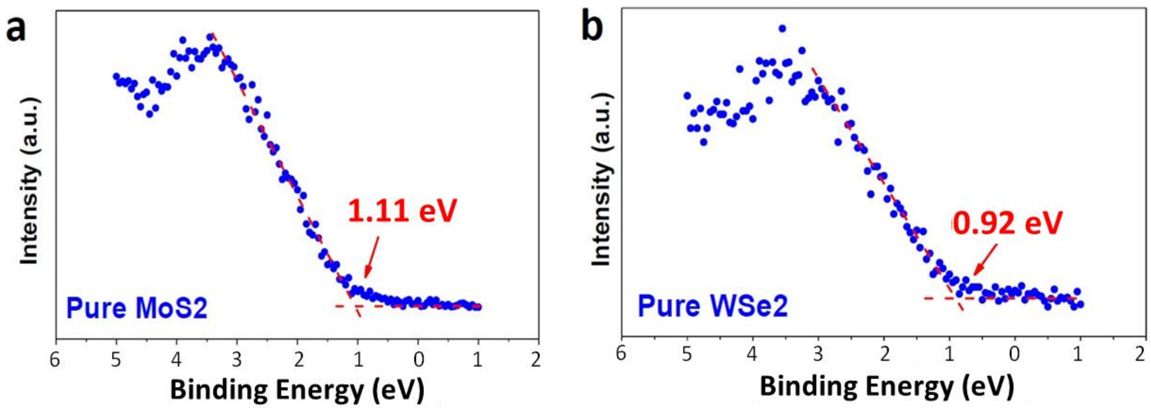

Supplementary Figure 2 | Determinations of VBM* using XPS

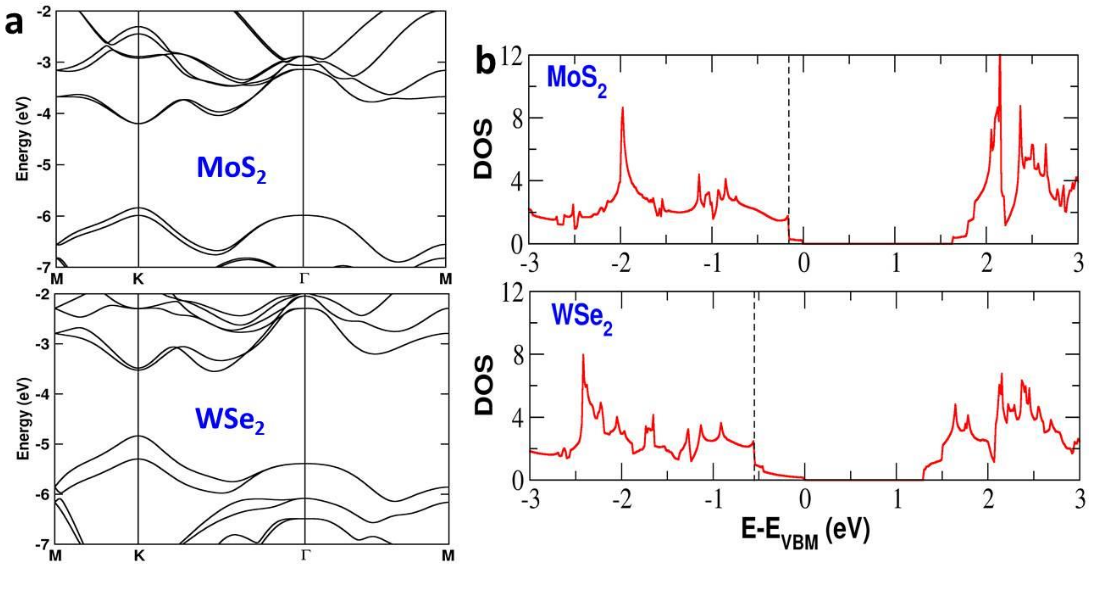

Supplementary Figure 3 | Theoretical calculation of band diagrams and DOS for MoS_2 and WSe_2 using DFT

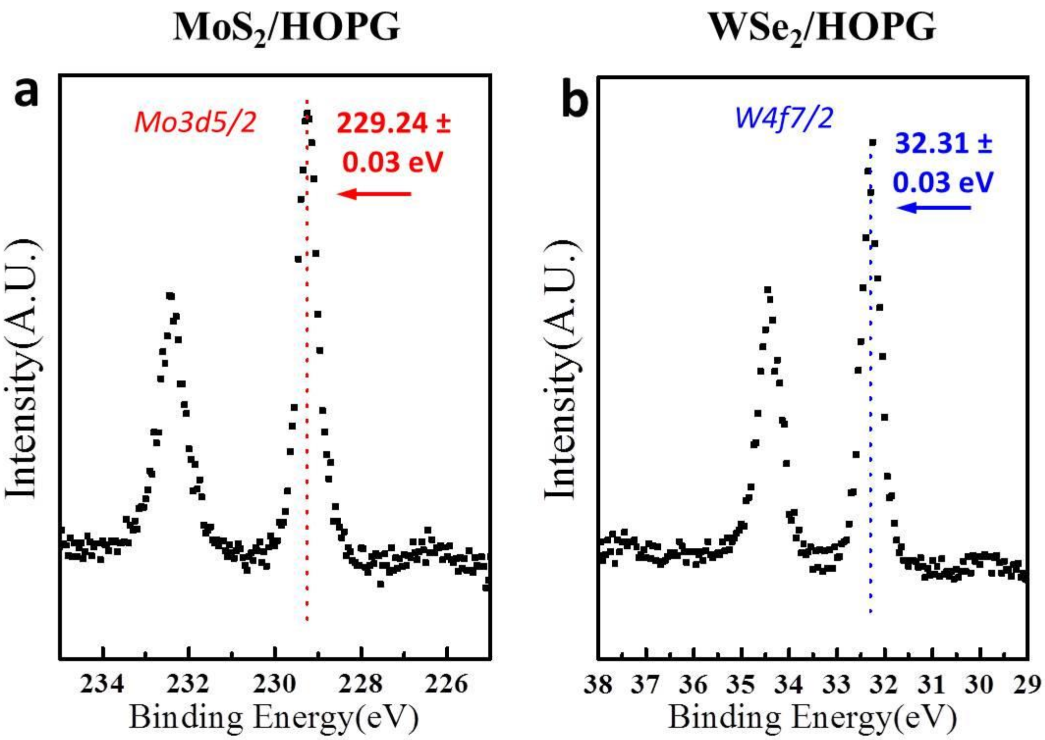

Supplementary Figure 4 | The μ-XPS taken on the SL MoS_2 and WSe_2 grown on HOPG. The measurements yield a binding energy of 229.24 ± 0.03 eV for $Mo3d_{5/2}$ (shown in **a**), corresponding to a separation of 228.40 eV to the true VBM (-1.84 eV measured by STS) in MoS_2. Similarly $W4f_{7/2}$ in SL-WSe_2 on graphite has a binding energy of 32.31 ± 0.03 eV (shown in **b**), corresponding to a value of 31.26 eV when referenced to the true VBM (-1.05 eV measured by STS).

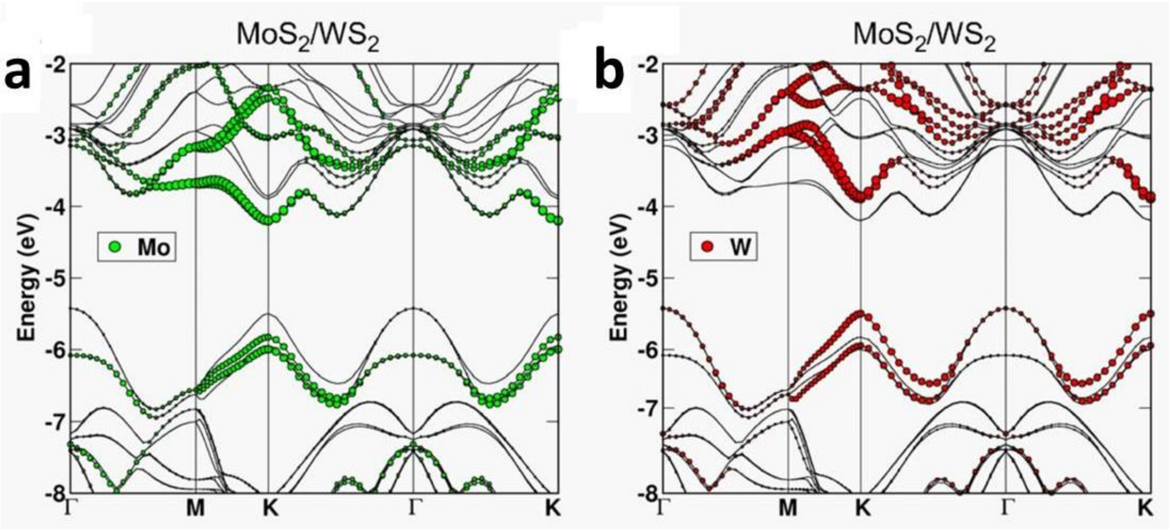

Supplementary Figure 5 | Theoretical calculations for electronic structure of the MoS_2/WS_2 bilayer

Supplementary Table 1

	Exfoliated			CVD grown		
	Raman (1/cm)		PL (eV)	Raman (1/cm)		PL (eV)
	E_{2g}	A_{1g}		E_{2g}	A_{1g}	
MoS_2	384	403	1.9	384	403	1.85
WSe_2	249	261	1.65	248	259	1.63

Summary of Raman and PL measurements for exfoliated and CVD grown SL-TMDs.

Supplementary Notes

Supplementary Note 1 | Determinations of VBM* using XPS

Supplementary Figure 2 illustrates how the VBM location is determined using XPS. Here the leading edge is extrapolated to intersect with the background base line. This procedure has been used to deduce the VBM position of conventional semiconductors whose VB DOS have very similar shape. The result is consistent with a more sophisticated least square fit with respect to broadened DOS. This procedure, however encounters some difficulties in determine the actual VBM position in TMD materials. In SL-TMDs, the global VBM is located at the K point.

However near the VBM, the DOS is dominated by the states near the Γ point (at the zone center) as illustrated in Supplementary Figure 3b which contains the theoretical calculation of DOS of two TMDs (MoS_2 and WSe_2) using DFT. The three dashed vertical lines mark the energy

positions of Γ points in three SL-TMD compounds respectively. The much larger contribution of DOS near the Γ point in comparison to that near the K point is due to a larger effective mass at the Γ point (by a factor of 4-5) and the double degeneracy. Since XPS cannot resolve the Γ-K splitting, the fitting of leading edge will consistently yield a VBM position very close to the local VBM at the Γ point, but offset by ~ 0.2 eV above it. Since VBO is referred to as the difference between the VBM positions, this offset will be cancelled out when VBO is deduced. Thus, the VBO deduced from the XPS measurement will correspond to the band offset of the local VBM at the Γ point (referred to as VBO*). As discussed in the main text, STS allows us to resolve Γ-K splittings which can be added to the VBO* to obtain accurate values of VBOs.

Supplementary Note 2 | Theoretical calculations for MoS_2/WS_2

The electronic structure of the MoS_2/WS_2 bilayer exhibits additional features as shown in Supplementary Figure 5 where the amount of Mo (W) projection is represented by the size of green (red) circles. The two layers have same chalcogen atoms and almost identical lattice constants. We have performed the calculations for a few stacking patterns and found that the band offset at the K point remains well defined and appears to be independent of the stacking pattern. However, the interlayer coupling moves the VBM position in the WS_2 layer from K to Γ point, creating an indirect gap about 0.1 - 0.2 eV smaller than the direct gap [Supplementary reference 1]. Note that our calculation of WS_2 on MoS_2 corresponds to a 60-degree (2H) stacking, lattice matched bilayer (namely an anti-aligned stacking). We note that a recent optical study of the MoS_2 bilayer system with artificial designed stacking angles shows that the interlayer coupling depends strongly upon the twist angle, with the coupling strength significantly reduced at twist angles between 0 and 60 degree [Supplementary reference 2]. Since

in our system of WS_2 on MoS_2, the VBM at the Γ point is about 0.1 - 0.2 eV above the K-point in contrast to a value of 0.4 - 0.5 eV for the bilayer, it remains to be seen whether the weakened interlayer coupling in the incoherent stacking will recover the direct gap characteristics.

Supplementary Note 3 | Calculation details

To account for the noticeable spin-orbit splitting in the energy bands of these transition metal dichalcogenides, relativistic effects are included in the calculation. The PAW potentials are generated by fully relativistic calculations for the free atom or ion. Keeping the relativistic effect up to the second order of the fine-structure constant, the Hamiltonian contains the scalar relativistic potential (including the mass-velocity and Darwin corrections) and the spin-orbit coupling term. Both of them were included in the self-consistent calculations for the extended double-layer systems. The calculation of the MoS_2/WSe_2 bilayer uses a supercell in which a $\sqrt{13} \times \sqrt{13}$ unit cell of MoS_2 and a $\sqrt{12} \times \sqrt{12}$ unit cell of WSe_2 are rotated by 16.1° with respect to each other. The resulting lattice mismatch is smaller than 0.2%. The interlayer separation is at 6.67 Å as determined by including the vdW-DF corrections. The plane-wave energy cutoff is 600 eV. The k-point sampling is 12×12×1 for the 1×1 unit cell and 4×4×1 for the supercell. A vacuum of 25 Å is used to eliminate the spurious interaction. The structure is fully relaxed until the change of the energy and the force reaches 10^{-6} eV per 1×1 cell and 10^{-2} eV/Å, respectively.

Supplementary References

1. Komsa, H. P. & Krasheninnikov, A. V. Electronic structures and optical properties of realistic transition metal dichalcogenide heterostructures from first principles. *Phys. Rev. B* **88,** 085318 (2013).
2. van der Zande, A. M. et al. Tailoring the electronic structure in bilayer molybdenum disulfide via interlayer twist. *Nano Lett.* **14**, 3869-3875 (2014).
